# Influence of a quality improvement intervention on rehabilitation outcomes of children (6–24 months) with acute malnutrition: a retrospective study in rural Angola

**DOI:** 10.1186/s12887-022-03585-8

**Published:** 2022-09-08

**Authors:** Andrea Pietravalle, Alessandro Baraldi, Martina Scilipoti, Francesco Cavallin, Magda Lonardi, Ivo Makonga Tshikamb, Claudia Robbiati, Daniele Trevisanuto, Giovanni Putoto

**Affiliations:** 1 Doctors With Africa CUAMM, Padua, Italy; 2 Doctors With Africa CUAMM, Luanda, Angola; 3 Independent Statistician, Solagna, Italy; 4 Missionary Catholic Hospital of Chiulo, Ombadja Municipality, Techiulo, Angola; 5 Department of Woman’s and Child’s Health, University of Padua, Padua, Italy

**Keywords:** Acute malnutrition, Nutritional rehabilitation, Default

## Abstract

**Background:**

Defaulting is the most frequent cause of Community Management of Acute Malnutrition (CMAM) program failure. Lack of community sensitization, financial/opportunity costs and low quality of care have been recognized as the main driving factors for default in malnutrition programs. The present study aimed to evaluate if a logistic reorganization (generic outpatient department, OPD vs dedicated clinic, NRU) and a change in management (dedicated vs non dedicated staff) of the follow-up of children between 6 and 24 months of age with acute malnutrition, can reduce the default, relapse and readmission rate and increase the recovery rate.

**Methods:**

Retrospective observational study on the impact of quality improvement interventions on rehabilitation outcomes of children (6–24 months) with acute malnutrition, admitted at the Catholic Mission Hospital of Chiulo (Angola) from January 2018 to February 2020. Main outcome measures were recovery rate, the default rate, the relapse rate, and the readmission rate.

**Results:**

The intervention was associated with a decrease in the default rate from 89 to 76% (*p* = 0.02). Recovery rate was 69% in OPD and 88% in NRU (*p* = 0.25). Relapse rate was nil.

**Conclusions:**

The present study supports the hypothesis that an improvement in quality of care can positively influence the rehabilitation outcomes of malnourished children. Further studies are needed to identify children at risk of low adherence to follow-up visits to increase the effectiveness of rehabilitation programs.

## Introduction

The term malnutrition refers to both under-nutrition and over-nutrition, but it is generally used to indicate undernutrition, including acute (wasting), chronic (stunting) and composite form depending on the extent and timing of nutritional deprivation. over 45% of all deaths among under-5 children has undernutrition as underlying cause. In addition the impaired development and growth in survivors often lead to adverse consequences in later life regarding intellectual ability, school achievement, work productivity and earnings [1]. In 2017, acute malnutrition affected worldwide nearly 51 million of under-5 children, with over 25% of them living in Africa [2]. Undernutrition has multifactorial genesis including environmental degradation, natural disasters, political and civil conflicts, poverty, infectious disease, inadequate access to food and feeding practices [3]. The last national census showed that in Angola, 38% of under-5 children are stunted and 5% are affected by wasting. Exclusive breastfeeding is 62% at 1 month of age and 17% at 4–5 months of age, and usually lasts around 3 months. The WHO minimum acceptable diet standard is met by only 13% of children aged 6–24 months. Under-5 mortality rate ranges from 68 to 98 deaths per 1,000 live births in urban and rural areas, varying by residence, province and household wealth [4].

Community Management of Acute Malnutrition (CMAM) program is the globally endorsed approach for the treatment of moderate and severe acute malnutrition in emergency and non-emergency settings. It works identifying malnourished children at community level and referring them to outpatient or hospital care in accordance with the severity of malnutrition [5]. The key indicators to define CMAM program success are represented by a recovery rate above 75%, a death rate below 10% and a default rate below 15% [6]. Defaulting seems to be the most frequent reason of CMAM program failure and is associated with lack of community sensitization (awareness about the programme and malnutrition), financial/opportunity costs (carer busy or sick, distance, lack of money) and inadequacies associated with the quality of care [7, 8].

The presence of under-resourced, overburdened, not adequately trained and unmotivated staff, necessarily leads to poor quality of care. A limited explanation of the child’s condition and its treatment may result in a failure to convey essential information which would encourage mothers to comply with weekly attendance until recovery [7, 8].

The present study aimed to evaluate if a logistic reorganization (generic outpatient department vs dedicated clinic) and a change in management (non dedicated vs dedicated nursing staff) of the follow-up of children between 6 and 24 months of age with acute malnutrition, can reduce the default, relapse and readmission rate and increase the recovery rate.

## Materials and methods

This is a retrospective observational study on the impact of quality improvement interventions on rehabilitation outcomes of children (6–24 months) with acute malnutrition, admitted at the Catholic Mission Hospital of Chiulo (Angola) from January 2018 to February 2020. The Ethics Committee of the Angolan Ministry of Health approved the study (ref. number 032020) and waived the requirement for written informed consent because of the retrospective study design and the use of anonymized data from hospital records. All procedures were performed in accordance with the relevant guidelines and regulations.

### Community Management of Acute Malnutrition (CMAM) program

CMAM program works identifying malnourished children at community level and referring them to Stabilization Centers (SC) or Outpatient Treatment Programs (OTP) in accordance with the severity of malnutrition. Children with severe (SAM) or moderate (MAM) acute malnutrition and medical complications are admitted to SC and receive F75 and F100 therapeutic milks. After stabilization of their clinical conditions and resolution of the complications (usually four to seven days), the treatment is carried on in the Outpatient Treatment Units (OTU) until nutritional recovery. Relapsed children are referred again to the SC. Children with SAM/MAM without medical complications receive routine medical treatment and nutrition rehabilitation with Ready to Use Therapeutic or Supplementary Foods (RUTF/RUSF) at the OTP. Children attend outpatient care at regular intervals (every one or two weeks) until recovery is achieved (usually two months) [5, 9].

### Setting

The Hospital of Catholic Mission of Chiulo (Cunene province, Angola) is a district hospital implementing the CMAM program in a rural area of 12,263 km^2^ with 345,490 inhabitants (including 60,392 under-5 children) [9]. As part of a network of 36 healthcare facilities involved in the national nutrition program, Chiulo Hospital acts as SC for the inpatient care of malnourished children with complications, as well as Outpatient Treatment Unit (OTU) for the rehabilitation phase after discharge. The nutritional rehabilitation unit counts 10 beds and is managed by a dedicated staff of doctors, nurses and paramedics. In 2018, 253 admissions were registered at the nutritional rehabilitation unit.

### Patients

All children aged 6–24 months with SAM/MAM discharged from SC were eligible for inclusion.

### Outcome measures

The outcome measures included the recovery rate, the default rate, the relapse rate, and the readmission rate. Definitions are provided in Sect. 2.6.

### Data collection

Children data included sex, age, severe/moderate malnutrition (as defined in Sect. 2.6), distance from Chiulo hospital, discharge information (in-hospital death, self-discharge, discharge) and follow-up data (default, readmission, attendance to follow-up visits, recovery and relapse) All data were retrospectively collected from hospital charts.

### Definitions

Malnutrition was defined by the combination of clinical assessment and anthropometric measurements (Weight for Height ratio or Mid-Upper Arm Circumference) according to WHO classification [10]. MAM was indicated by Weight for Height ratio ≥ 3 and < 2 Standard Deviation, or Mid-Upper Arm Circumference > 115 and < 124 m. SAM was indicated by Weight for Height ratio < 3 Standard Deviation and Mid-Upper Arm Circumference ≤ 115 mm. Within the CMAM program, an admission is the first contact with the program; a default designates a beneficiary who is absent for two consecutive weightings; a relapse designates a beneficiary readmitted to the program after having been successfully discharged as recovered within the last two months; a readmission identifies a beneficiary readmitted to the program within two months of leaving it for a reason other than recovery (e.g. defaulting or non-response) [4]; a recovery designates a beneficiary achieving weight-for-height ratio ≥ -2 Standard Deviation at least or Mid-Upper-Arm Circumference ≥ 125 mm and without edema for at least 2 weeks [5].

### Comparisons

A comparative analysis was carried out to assess the impact of the quality improvement intervention on rehabilitation outcomes of children (6–24 months) with acute malnutrition. The intervention included the introduction of a dedicated clinic for the rehabilitation follow-up of the malnourished children. This clinic was located within the SC in the Nutritional Rehabilitation Unit (NRU) and was managed by dedicated and adequately trained staff.

**Fig. 1:**
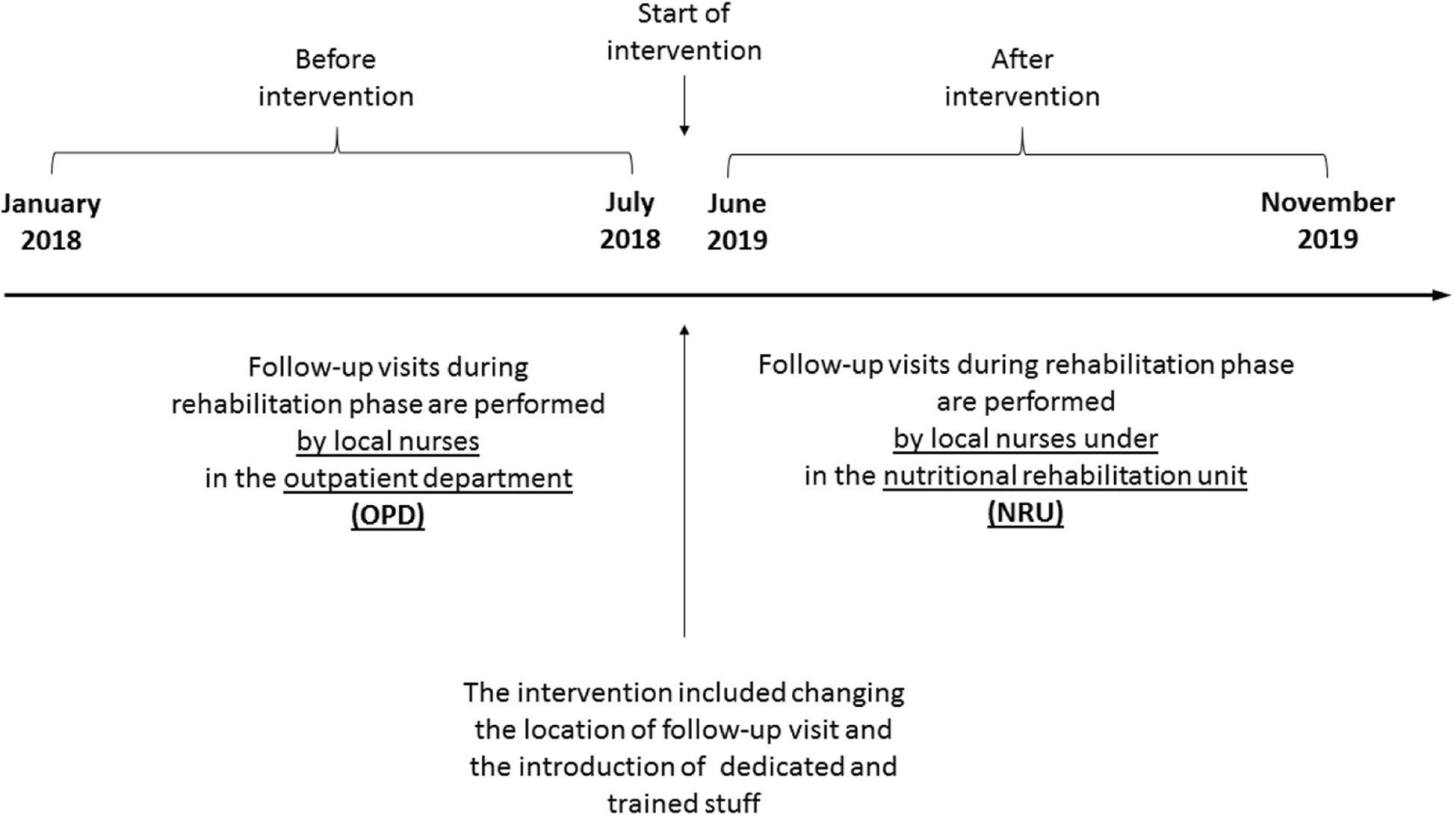
Scheme of intervention

In the main analysis, we compared the 6-month period following the introduction of this clinic (June-November 2019) with a previous 6-month period (January-July 2018) when the follow-up was managed in Out Patient Department (OPD), which was not exclusively dedicated to malnourished patients but responsible for evaluating all accesses for pediatric visits (Fig. 1). The choice of comparing these non-consecutive periods was due to ensure that both groups under comparison had received RUTF/RUSF.

In a first sub-analysis, we compared two sub-periods of 4 months (April-July 2018 vs August-November 2019), in which the caregivers had superimposable work commitment in agriculture activities. The purpose was to limit the influence of financial/opportunity costs on the default rate.

In a second sub-analysis, we compared the two 6-month periods in the subsets of patients living in the Chiulo area. The purpose was to limit the effect of the distance from the treatment center, which could affect the outcome of interests.

### Statistical analysis

Categorical data were summarized as frequency and percentage, and continuous data as median and interquartile range (IQR). Child characteristics were compared between OPD and NRU using Chi Square test and Mann–Whitney test. Outcome measures were compared between OPD and NRU using Chi Square test and Fisher’s exact test (unadjusted analysis). The comparison of default between OPD and NRU was also adjusted for unbalanced characteristics at baseline (age and SAM/MAM) and distance from the treatment center using a logistic regression model (adjusted analysis). The small sample size at follow-up visits and in the subset of patients living in Chiulo area did not allow any meaningful multivariable analyses. The association between default rate and distance from Chiulo was evaluated using a Beta regression model using data from 2018–2019. All tests were 2-sided and a p-value below 0.05 was considered statistically significant. Statistical analysis was performed using R 4.0 (R Foundation for Statistical Computing, Vienna, Austria) [11].

## Results

Overall, 402 children (128 in Jan-Jul 2018 and 274 in Jun-Nov 2019) were include in the study. Children characteristics are shown in Table 1. Children admitted to NRU (June-November 2019) were older (*p* = 0.001) and with a larger proportion of SAM (*p* < 0.0001) compared to those admitted to OPD (January-July) (Table 1).

**Table 1:** Child characteristics

	**January-July 2018**	**June-November 2019**	***p*-value**
**N children**	128	274	-
Males, n (%) Females, n (%)	58 (45.3) 70 (54.7)	137 (50.0) 137 (50.0)	0.44
Age (months), median (IQR)	12 (9–12)	12 (10–17)	0.001
SAM, n (%) MAM, n (%)	83 (64.8) 45 (35.2)	230 (83.9) 44 (16.1)	< 0.0001
Distance from Chiulo (km), median (IQR)	29 (14–39)	29 (16–58)	0.08

**Fig. 2:**
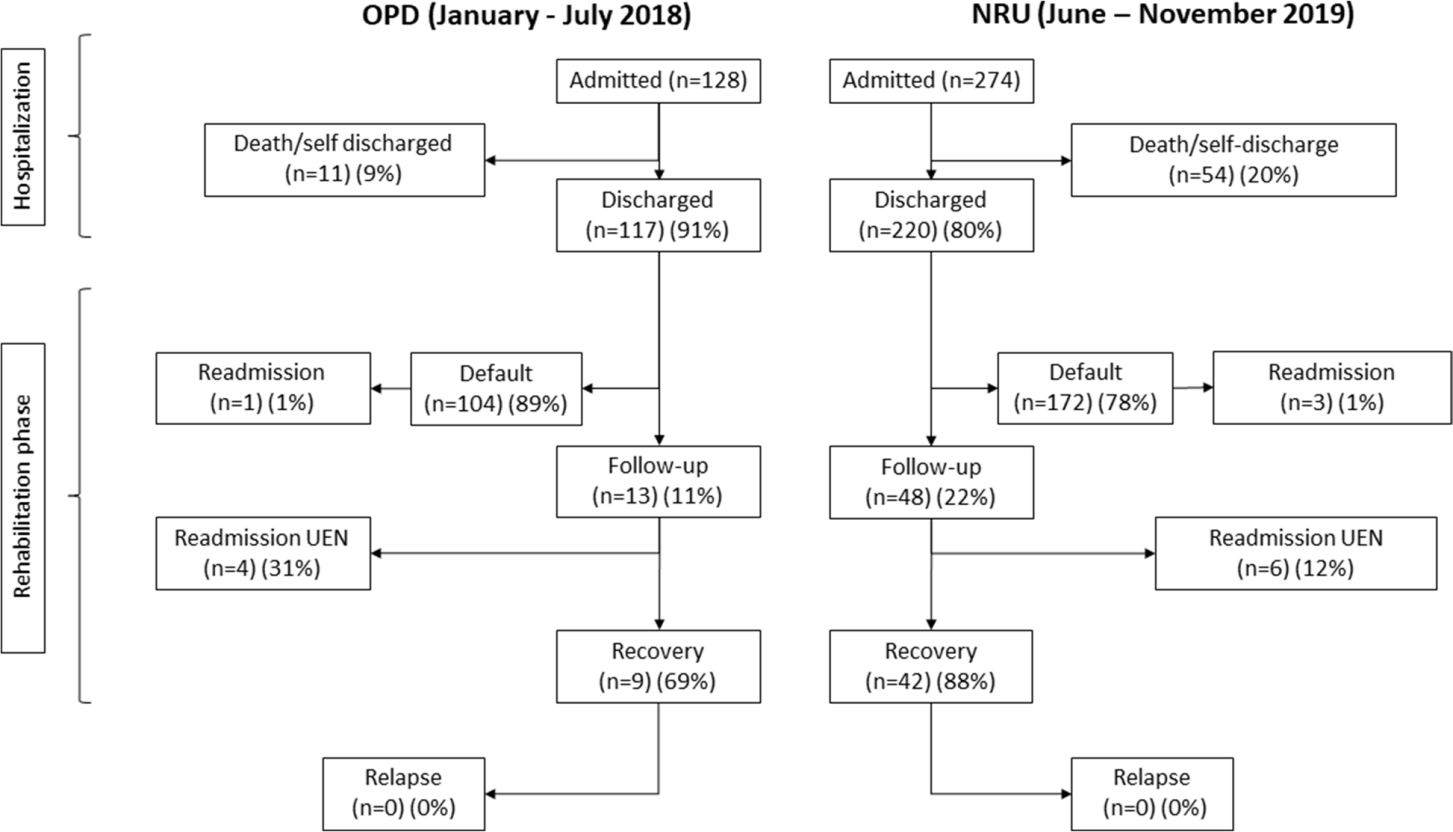
Flow-chart of patients during follow-up (OPD January-July 2018 vs. NRU June-November 2019)

In the main comparison (OPD January-July 2018 vs. NRU June-November 2019), default rate decreased from 89% (104/117) in OPD to 76% (143/189) in NRU (*p* = 0.02) (Fig. 2). Multivariable analysis confirmed the reduced default rate in NRU vs. OPD (*p* = 0.01) and showed increased default rate with increasing distance from Chiulo (*p* = 0.0003) (Table 2). Recovery rate was 69% (9/13) in OPD and 88% (42/48) in NRU (*p* = 0.25), while relapse rate was nil (Fig. 2).

**Table 2:** Multivariable analysis of default

Comparison	Variable	Odds ratio (95% confidence interval)	*p*-value
**OPD January-July 2018** **vs** **NRU June-November 2019**	NRU vs. OPD	0.38 (0.18 to 0.77)	0.01
	Age at admission, days	1.01 (0.96 to 1.07)	0.63
	SAM vs. MAM	0.90 (0.42 to 1.83)	0.92
	Distance from Chiulo, km	1.03 (1.1 to 1.05)	0.0003
**OPD April-June 2018** **vs** **NRU August-November 2019**	NRU vs. OPD	0.34 (0.12 to 0.81)	0.03
	Age at admission, days	1.00 (0.95 to 1.06)	0.91
	SAM vs. MAM	0.90 (0.38 to 1.97)	0.79
	Distance from Chiulo, km	1.03 (1.01 to 1.05)	0.002

**Fig. 3:**
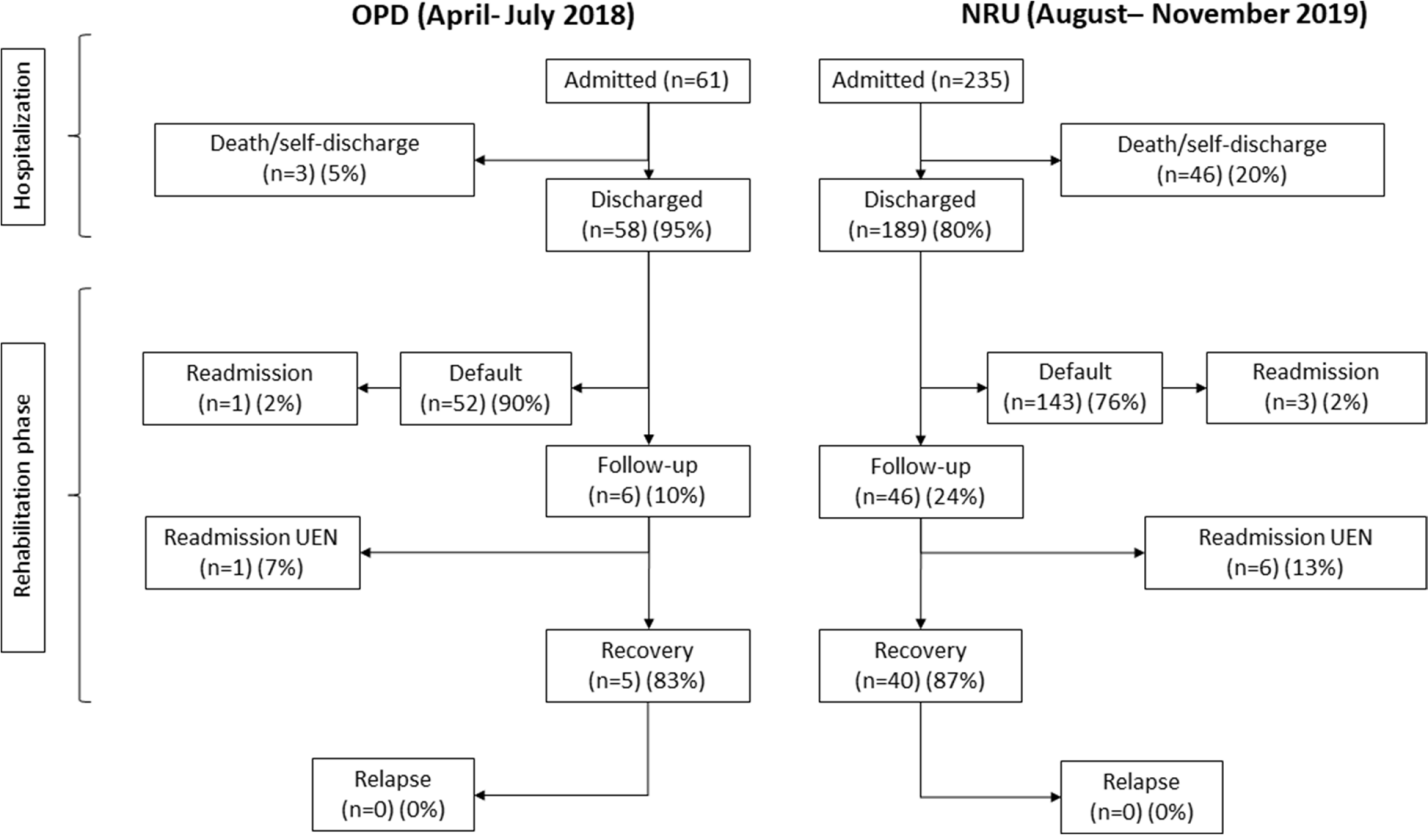
Flow-chart of patients during follow-up (OPD April-July 2018 vs. NRU August-November 2019)

In the first sub-comparison (OPD April-July 2018 vs NRU August-November 2019), default rate decreased from 90% (52/58) in OPD to 76% (143/189) in NRU (*p* = 0.03) (Fig. 3). Multivariable analysis confirmed the reduced default rate in NRU vs. OPD (*p* = 0.03) and showed increased default rate with increasing distance from Chiulo (*p* = 0.002) (Table 2). Recovery rate was 83% (5/6) in OPD and 87% (40/46) in NRU (*p* = 0.99) (Fig. 3).

**Fig. 4:**
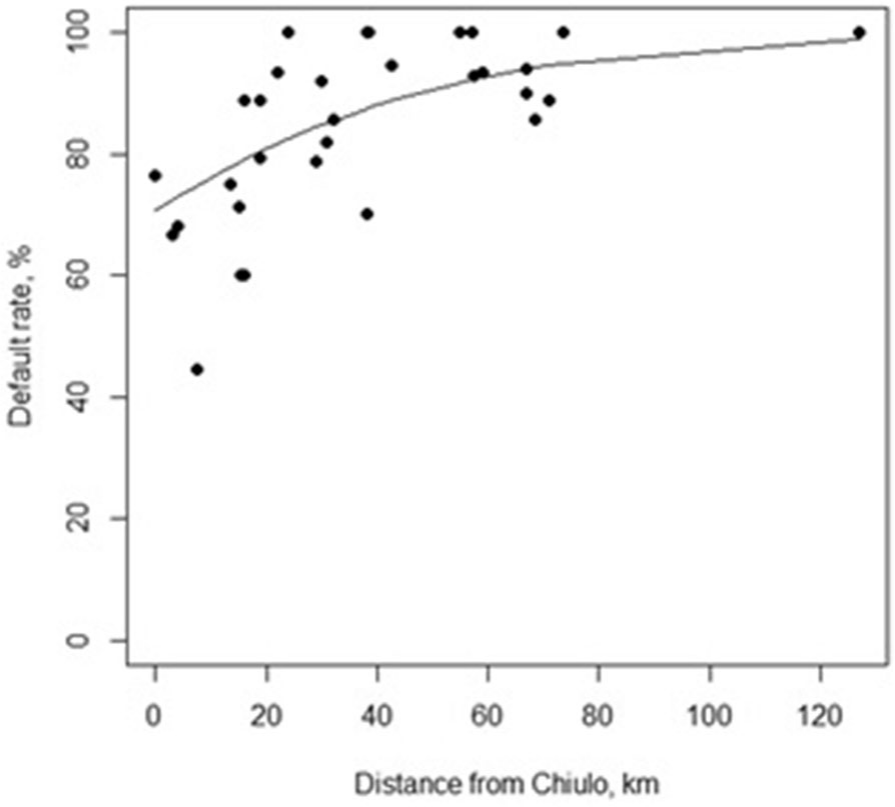
Association between default rate and distance from Chiulo

**Fig. 5:**
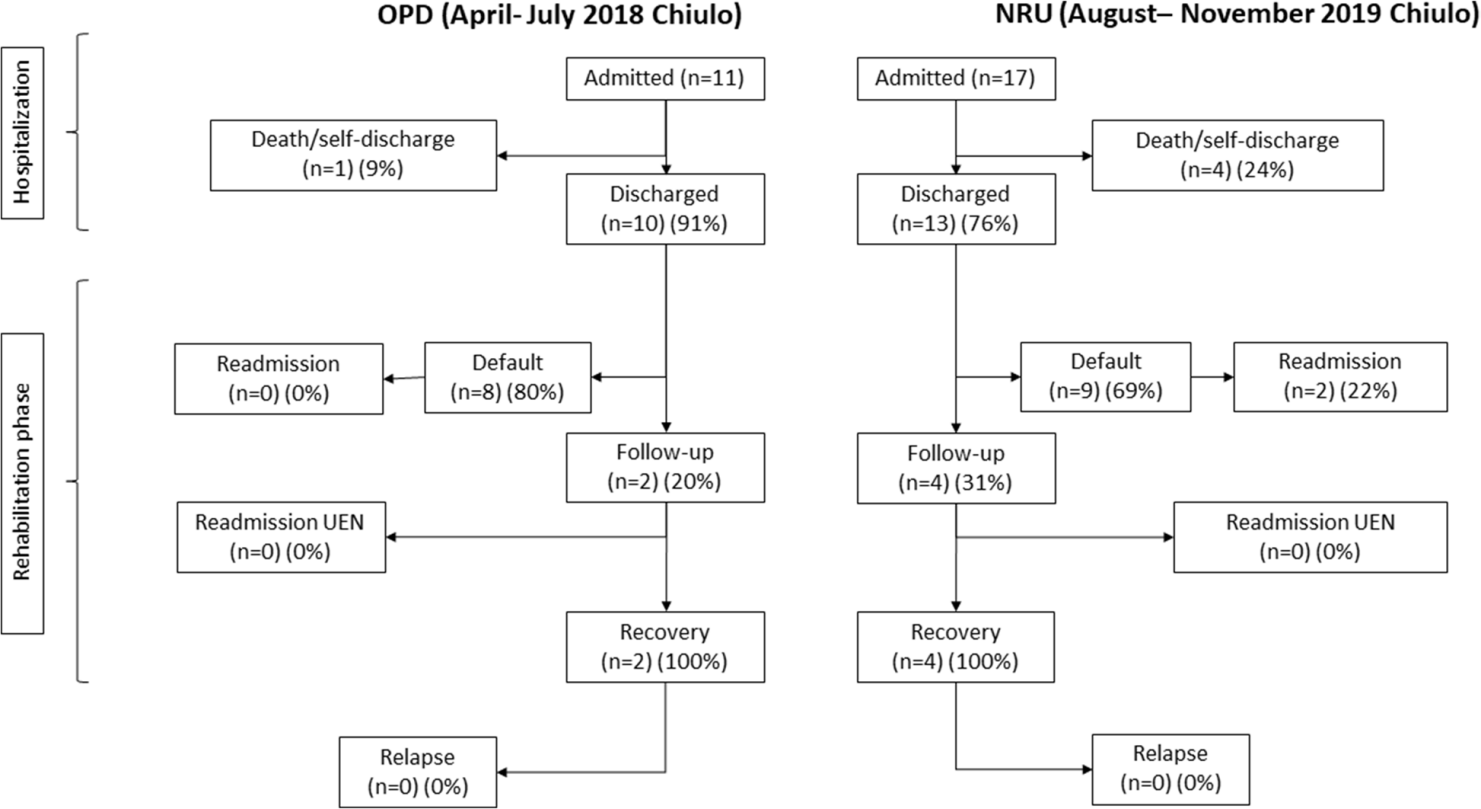
Flow-chart of patients living in Chiulo area during follow-up (OPD January-June 2018 vs. NRU June-November 2019)

In 2018–2019 period, default rate increased with longer distance from Chiulo (*p* < 0.0001) (Fig. 4). When restricting the analysis to patients living in Chiulo area, default rate was 80% (8/10) in OPD and 69% (9/13) in NRU, while recovery rate was 100% (2/2) in OPD and 100% (4/4) in NRU (Fig. 5). Statistical testing was not performed due to the small sample size.

## Discussion

Our findings showed a significant reduction of default rate after the introduction of a dedicated clinic for the rehabilitation follow-up of the malnourished children. Nonetheless, default rate remains high and limited further improvements in recovery rate and relapse rate.

In the treatment of acute malnutrition, about 24% of the default cases can be ascribed to inadequacies in the quality of care [7]. Given the efforts made by caregivers to access treatment, the environment of care, the communication with health care providers and the information received during the treatment can become a key factor for their continuing attendance [8]. Staff/beneficiary ratios and the corresponding time that staff can allocate to each service user can be targeted by interventions for improving the quality of the interface between SAM treatment services and caregivers. In fact, CMAM program services often rely on overburdened community-based health workers [12]. The service delivery model funnels all SAM cases towards a limited number of facilities, forcing these under-resourced, overburdened and often demotivated health workers to deliver treatment to a high number of cases per week [8]. This not only increases the waiting times [13] but also decreases the amount of time available for communicating with each caregiver [8]. The lack of adequate health workers training and homogenization between the referral and admission criteria, result in a high number of case rejections [14]. An inadequately explained rejection results in frustration which in turn prevents future attendance when the child’s condition deteriorates [15]. Available literature suggests that rejection and staff/beneficiary interface can be positively influenced by supervision and motivation [16]. Our findings support such consideration confirming the key role of providing dedicated, adequately trained, and motivated staff.

Among the financial/opportunity costs (which may be responsible for about 25% of the default cases), distance from health facility plays an important role [7, 8]. Despite the advantages of the decentralization of SAM management from hospitals to health facilities, the access to treatment still implies travelling significant distances for most caregivers [17]. Early community-based SAM treatment models clearly specified the need to place service delivery points within 3-h walk (one day round-trip) of the targeted populations or within a 15 km threshold [18]. Our data confirmed the association between longer distance and higher default rate. This issue is exacerbated by the actual travel distance, as most caregivers lived over 20 km from the hospital and usually travelled on foot due to economical restrictions. Nonetheless, default rate was very high (around 70–80%) also among children living in Chiulo area, thus implying the effect of other factors such as lack of community sensitization. The awareness about the program and malnutrition has been identified as the most important cause of default (45% of all default cases) [7]. The main strategy to address this problem consists in raising awareness about the condition and the treatment services available, through the involvement of key community figures, including local leaders, Traditional Health Practitioners and community groups [19, 20].

A future intervention to improve community sensitization, may consider the introduction of specific educational sessions in the immunization service, routinely performed by the Public Health Staff of Chiulo Hospital during dedicated outpatient clinic in health centers and outreach sites.

Overall, this study suggests that introducing a dedicated clinic for the rehabilitation follow-up of the malnourished children may reduce the default rate. However, the reader should be aware of the study limitations. The retrospective design limited the availability of potentially important data such as background information, possible confounders, and reasons for not attending follow-up visits. Such data can be retrieved in prospective studies using pre-established data collection forms and including contacts with caregivers of children not compliant to follow-up visits. The identification of children at risk of low adherence to follow-up may increase the effectiveness of the rehabilitation program. The single-center design limited the available sample size for analysis and the generalizability of the findings to similar settings. Implementing the interventions in multiple sites may help in overcoming such limitations. Lastly, we cannot exclude that the inclusion of different months in before-intervention and after-intervention periods might have introduced some seasonality-related effects on the findings. Unfortunately, the choice of the periods for comparison was influenced by the retrospective design, the timing of the intervention, and the need for ensuring that RUTF/RUSF was available in both periods under comparison. In the analysis, we also considered the impact of seasonality on workload, and we performed a sub-analysis comparing two sub-periods of 4 months (April-July 2018 vs. August-November 2019), in which the caregivers had superimposable work commitment in agriculture activities.

## Conclusions

The data emerging from the present study support the hypothesis that an improvement in quality of care can positively influence the rehabilitation outcomes of malnourished children. Further studies are needed to identify children at risk of low adherence to follow-up visits in order to increase the effectiveness of rehabilitation programs.

## Data Availability

The datasets used and/or analyzed during the current study are available from the corresponding author on reasonable request.

## Abbreviations

RUTF/RUSF: Ready-to-Use Therapeutic /Supplementary Food
CMAM: Community Management of Acute Malnutrition
SAM: Severe Acute Malnutrition
MAM: Moderate Acute Malnutrition
SC: Stabilization Center
OTP: Outpatient Treatment Program
OTU: Outpatient Treatment Unit
OPD: Outpatient Department
NRU: Nutritional Rehabilitation Unit
